# A systematic mapping review of qualitative research in paediatric otolaryngology

**DOI:** 10.1017/S0022215125104015

**Published:** 2026-03

**Authors:** Adam Mallis, Angus Lawson, Jason Powell

**Affiliations:** 1 Translational and Clinical Research Institute, Faculty of Medical Sciences, Newcastle University, Newcastle upon Tyne, UK; 2 Department of Paediatric Otolaryngology, Great North Children’s Hospital, The Newcastle upon Tyne Hospitals NHS Foundation Trust, Newcastle upon Tyne, UK

**Keywords:** otolaryngology, otorhinolaryngologic diseases, paediatrics, scoping review, review

## Abstract

**Objective:**

To map the scope, methods and focus areas of qualitative research in paediatric otolaryngology.

**Methods:**

A Preferred Reporting Items for Systematic Reviews and Meta-Analyses-compliant systematic mapping review searched MEDLINE, Embase, CENTRAL and PsycInfo (August 2025) for qualitative or mixed-methods studies with a qualitative component related to paediatric otolaryngology. Two reviewers independently applied the inclusion criteria. Key study characteristics were extracted; no formal risk-of-bias assessment was performed, in line with the aims of a mapping review.

**Results:**

Eighty-nine studies were included. Publications rose sharply after 2015, with nearly three-quarters from the USA, Canada and the UK. Otology (49 per cent) and laryngology (40 per cent) predominated; common topics were hearing loss, tonsillectomy and tracheostomy. Interviews, mainly semi-structured (73 per cent), were the dominant method, and caregivers were the most frequent participants (62 per cent).

**Conclusions:**

Qualitative research in paediatric otolaryngology is growing but remains geographically and methodologically narrow. Broader stakeholder inclusion and methodological diversity are needed to deepen understanding and support patient-centred care.

## Introduction

Quantitative methodologies have long constituted the preferred paradigm of research in medicine, with the randomised controlled trial being held up as the gold standard. Its advantages have included the ability to establish cause-and-effect relationships and define the certainty with which those relationships exist.^1^ Over recent decades, however, qualitative research has gained recognition as a complementary set of approaches, becoming more prominent across both medicine and other fields.^2^

It can be challenging to clearly define qualitative research, as there is no singular, agreed-upon set of criteria that delineates its scope. While techniques such as interviews, focus groups and surveys are often associated with qualitative research, they are not inherently qualitative and can just as easily be used to generate quantitative data.^3^ Rather, qualitative research is typically characterised by the forms in which data is collected, interpreted and analysed. Specifically, it involves ‘broadly stated questions about human experiences and realities, studied through sustained contact with the individual in their natural environments and producing rich, descriptive data that will help us to understand those individuals’ experiences’.^4^ Such data is commonly analysed through methods such as thematic analysis, narrative analysis or grounded theory, rather than traditional quantitative methods. As a result, qualitative research is especially suited to exploring ‘how’, ‘what’ and ‘why’ questions.^5^

Qualitative medical research has numerous unique advantages in medicine. Participant data is often detailed, complex, and guided to a greater extent by the subject’s opinions and perspectives than quantitative research often is. This enables a richer contextualisation of findings through a holistic lens with strong recognition of the patient voice.^6^ Moreover, the individualistic nature of qualitative data collection lends itself well to the creation of new distinctions and variables beyond those anticipated by researchers.^3^ These strengths are balanced, however, by drawbacks such as high resource demands, small sample sizes and consequently lower generalisability.^6^ In this sense, qualitative research often trades breadth for depth.

These advantages are particularly salient in otolaryngology, a field that deals not only with clinical outcomes but also with deeply personal aspects of the human experience, such as communication, breathing, eating and socialisation.^7^ Many of these experiences are subjectively felt by the patient in the context of their lives, families and cultures. As such, qualitative research is particularly important for understanding the perspectives of patients and those around them in relation to their care for otolaryngological conditions. This is especially true in paediatrics, where otolaryngological problems can affect communication and socialisation with lifelong consequences. Children are also deeply embedded in social networks, relying on parents or guardians who often act as both caregivers and advocates.

Accordingly, this review aimed to map out the landscape of existing qualitative literature in paediatric otorhinolaryngology.

## Materials and methods

### Inclusion and exclusion criteria

The systematic mapping literature review was conducted by two independent reviewers (AM and AL) in accordance with the Preferred Reporting Items for Systematic Reviews and Meta-Analyses (PRISMA) checklist.^8^

First, inclusion and exclusion criteria were developed. Inclusion criteria included:

- Original research studies including some qualitative technique(s) and analysis. It was not sufficient for a study to quantitatively analyse a subjective, ‘qualitative’ (often categorical) metric. Similarly, systematic reviews (qualitative or otherwise) were not included.
- Focus on paediatric otolaryngology, either wholly or partially, but with some distinct analysis relevant to that field.
- Published during or before July 2025.
- Has a PubMed ID.

Additional exclusion criteria included:

- Studies addressing only medical workforce or HR-related issues (e.g, factors influencing the assessment of residency applications).
- Studies not available in the English language. Unfortunately, it was not feasible to consider professional translation of non-English-language papers, which would likely have been required for appropriate analysis.^9^

For studies relating to certain topics, such as cleft palate, it was sometimes difficult to determine whether the research fell within the scope of paediatric otolaryngology. This ambiguity arises because, while cleft palate care is led by otolaryngologists in some centres, it is more commonly managed by specialists in other surgical disciplines. In order to maintain relevance and consistency, such studies were included only when they contained analysis specifically addressing the role or perspective of otolaryngology in the management of the condition.

Consistent with other systematic mapping reviews designed to characterise the research landscape, no inclusion or exclusion criteria were applied regarding study quality.^10^^–^^12^

### Literature search

Four databases were searched in August 2025: MEDLINE (via PubMed), Embase (via Elsevier), CENTRAL (via Cochrane Library), and PsycInfo (via Ovid). The search strategy was as follows: (paediatric* OR pediatric* OR child* OR newborn* OR neonat*) AND (ENT OR otolaryngology OR otorhinolaryngology OR otology OR laryngology OR rhinology) AND (qualitative OR interview OR focus group OR survey).

One thousand, three hundred and twenty-eight articles were initially identified and imported into Rayyan^13^ for manual processing. Following de-duplication, 39 duplicate articles were deleted, and 1,289 remained for screening.

**Figure 1.**
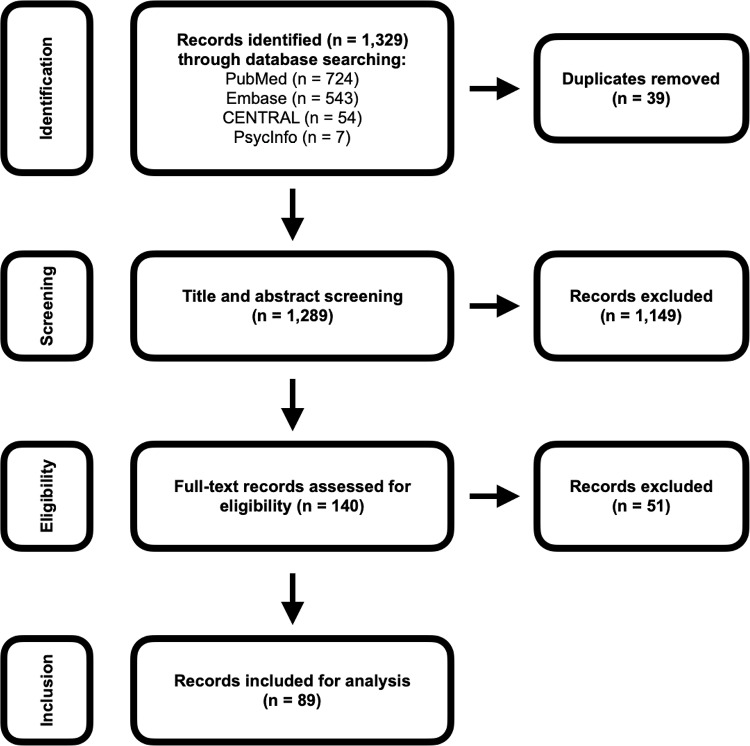
PRISMA flowchart.

Titles and abstracts were screened for relevance independently by two reviewers (AM and AL) who were blinded to each other’s decisions. Advice was also sought from JP in cases of discrepancy between the reviewers. At all stages, conflicts were resolved unanimously following discussion between the reviewers. A total of 140 articles progressed to full-text screening, of which 89 met all inclusion criteria.

### Data extraction

Relevant data points were extracted from the included articles. These included:

- Year of publication
- Country of origin
- Sub-specialty and topic area
- Methodology and subject of analysis
- Purpose of research

Where included studies utilised mixed-methods approaches, the above data points were extracted considering how they related to the qualitative portions of the research.

The process of categorising study aims, methods and participant groups was iterative and reflexive. Initial codes were generated inductively during data extraction, then refined through repeated engagement with the dataset. Categories were adjusted and, where necessary, combined to ensure they adequately captured overlap and diversity across studies. This iterative process is consistent with established approaches in qualitative synthesis, which emphasise flexibility and responsiveness to data.^14^

## Results and analysis

Many papers were assigned multiple codes within some categories of results. As such, percentages below represent the proportion of papers that were coded for a given item, rather than the relative frequency of that item across the total number of datapoints.

### Trends in time and geography

In a trend that mirrors broader patterns of change in qualitative research, the vast majority of included articles were from 2015 onwards, with only 10 per cent having been published earlier. Publication rates peaked in 2023 and 2024, and five papers were published across the first seven months of 2025. Though the sample size is relatively low, these data suggest a continuing upward trajectory in qualitative paediatric otolaryngology research.

**Figure 2.**
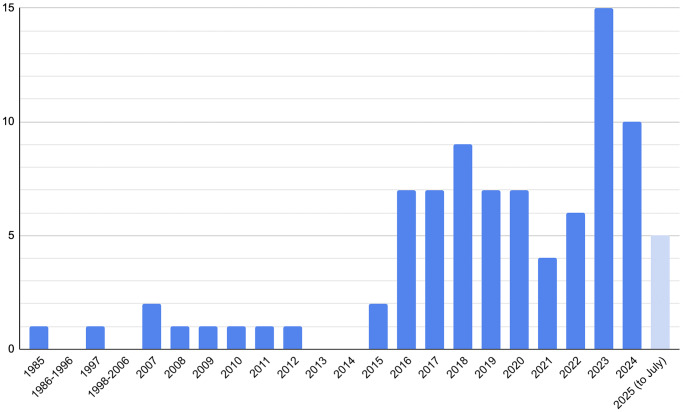
Studies by year published.

Geographical representation also varied considerably. North America accounted for 63 per cent of included articles, followed by Europe at 19 per cent.

**Figure 3.**
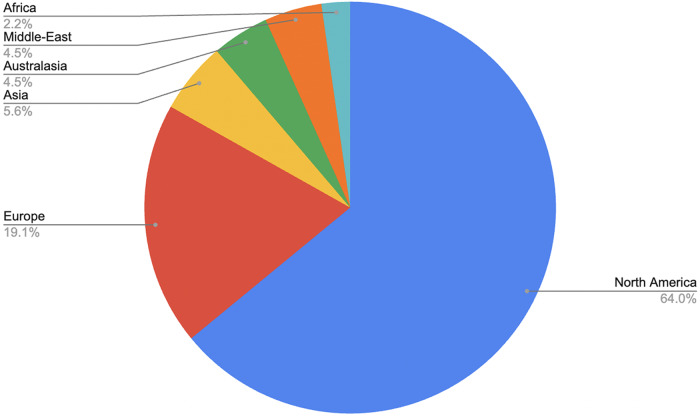
Studies by region.

At the national level, the United States contributed the largest share (47 per cent), followed by Canada (17 per cent) and the United Kingdom (13 per cent). No other individual nation represented more than 4 per cent of the sample. This distribution likely reflects the inclusion criteria requiring English-language publications.

**Figure 4.**
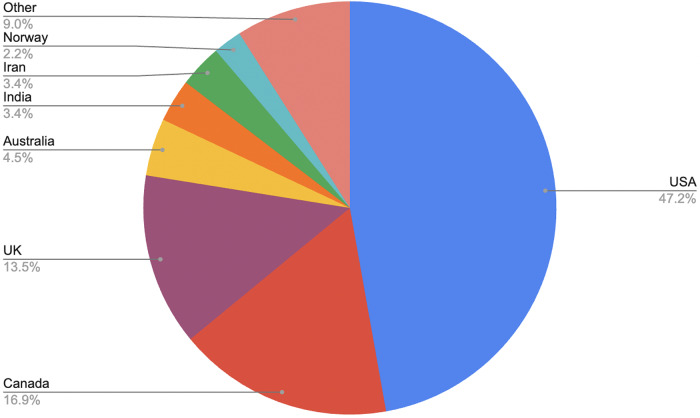
Studies by nation.

### Range of topics included in research

The scope of topics addressed by qualitative research in paediatric otolaryngology was broad. Nine per cent of the analysed articles explored issues across the breadth of paediatric otolaryngology. The most prevalent sub-specialty was otology (49 per cent), followed by laryngology (40 per cent).

**Figure 5.**
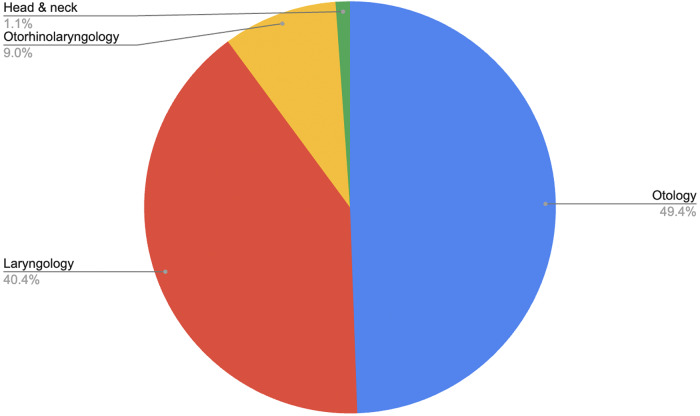
Studies by primary sub-specialty.

With respect to specific pathologies, the most common focus was general hearing loss of mixed aetiology (22 per cent), followed by tonsillectomy/adenotonsillectomy (13 per cent) and tracheostomy (13 per cent). Cochlear implantation (11 per cent) and sleep-disordered breathing (9 per cent) followed. No other pathology featured in more than 6 per cent of articles.

**Figure 6.**
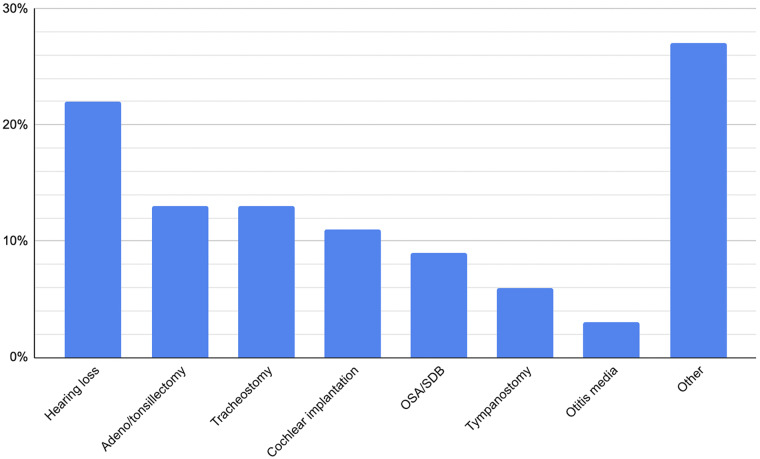
Percentage of studies including each pathology.

### Methods of included studies

Included articles were examined to determine both the qualitative methods used and the study participants (e.g. patients, otolaryngologists, etc). Thirteen per cent of studies made use of multiple qualitative methodologies, and 34 per cent obtained data from multiple categories of participants.

The most prevalent method among included papers was the use of interviews (73 per cent), followed by questionnaires/surveys (22 per cent), social media analysis (7 per cent) and focus groups (6 per cent). The most frequent methodological pairing was interview and questionnaire/survey (9 per cent). Among studies using interviews, semi-structured formats dominated (89 per cent).

**Figure 7.**
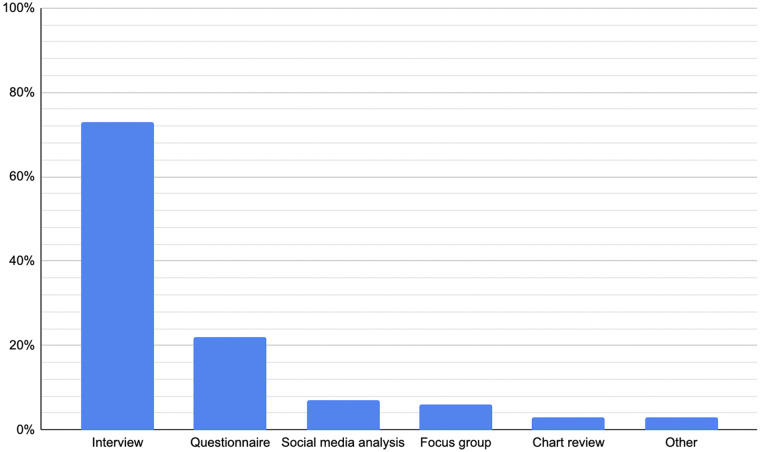
Percentage of included studies incorporating each method.

The most common study participants were families and primary caregivers (62 per cent), followed by non-otolaryngologist healthcare professionals (27 per cent), patients themselves (24 per cent) and otolaryngologists (13 per cent).

**Figure 8.**
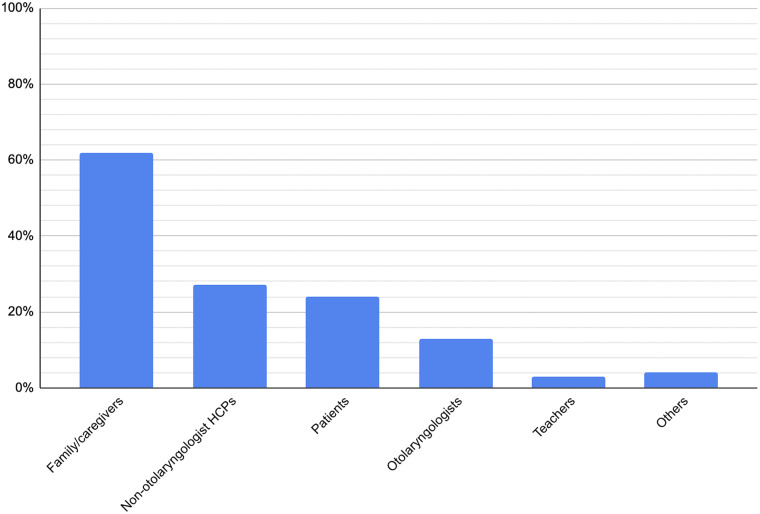
Percentage of included studies incorporating each participant group.

### Purpose of research

The primary aims of included studies were diverse. The most common objective was to examine barriers to accessing care (15 per cent). Other frequent aims included exploring the impact of illness on families and carers (12 per cent) and evaluating new tools or techniques (12 per cent). Additional frequently investigated aims included understanding stakeholder decision-making (11 per cent), developing outcome measures or other novel tools (10 per cent) and assessing service provision (9 per cent).

**Figure 9.**
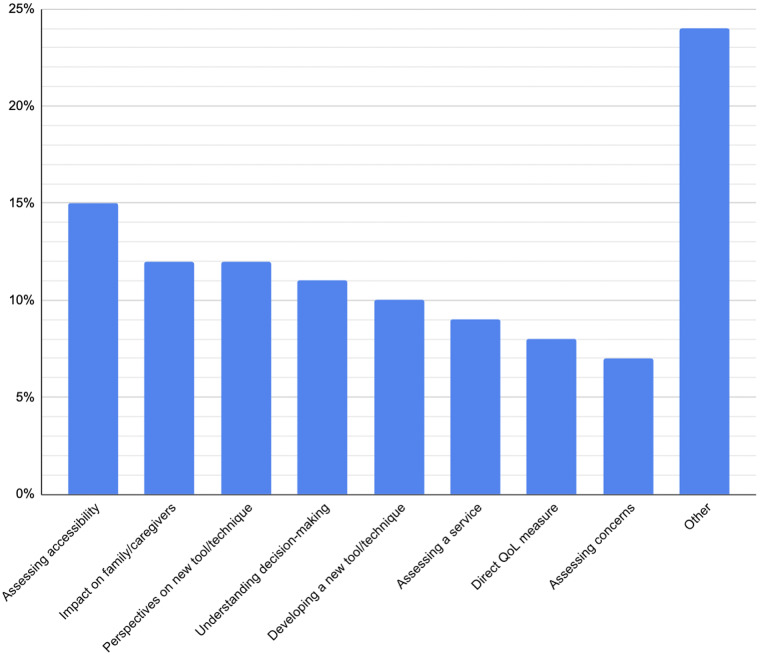
Percentage of studies with each objective.

## Discussion

### Trends in time and geography

The trend of increasing amounts of qualitative research in paediatric otolaryngology over time mirrors that of the growth in qualitative research more generally. This likely reflects the growing recognition of the value of such studies and the unique perspective that they can bring, as discussed in the introduction of this article.

The geographic distribution of included studies did not appear to change significantly over time. There was a consistent trend of the USA, Canada and the UK dominating the research output. This is likely at least partly a reflection of the English-language inclusion criterion,^15^ and may have led to a skewed dataset due to potential differences between English-language and non-English-language research. However, it also parallels the overall trend within medical research that the USA is the most significant contributor in most fields,^16^ with the UK and Canada frequently among the top five or top ten. The relative lack of included studies from prolific research-generating countries, such as China, Japan and many non-English speaking European countries, may reflect more on this review’s inclusion criteria than a lack of relevant research output.

### Range of topics included in research

This review highlights a diversity of topics addressed within qualitative research in paediatric otolaryngology. Though a proportion (9 per cent) of studies took a cross-sub-speciality perspective, most considered either one or a few pathologies, mostly across a single sub-speciality. The high prevalence of otology (44 per cent) likely reflects both the high clinical burden of hearing loss in childhood and the wide breadth of associated patient and family experiences that lend themselves to qualitative inquiry, including communication difficulties, educational challenges and psychosocial impacts. Similarly, the high representation of laryngology (40 per cent) suggests strong research interest in conditions such as airway compromise and vocal disorders, which have immediate implications for quality of life and require a nuanced understanding of patient and caregiver perspectives.

The distribution of research by pathology underscores these patterns further. General hearing loss of mixed aetiology was the single most common focus (22 per cent), emphasising the centrality of auditory health and hearing in paediatric speech and language development.^11^ Though there are some pathology-dependent differences in the experiences of children with hearing loss, there is also a significant amount of overlap in how the conditions impact their daily lives, which enables some qualitative research to consider them as part of a larger group.

Tonsillectomy/adenotonsillectomy accounted for 13 per cent of articles, and represent some of the most frequently performed paediatric surgeries worldwide, making them a natural area of interest for studies exploring patient experiences, decision-making and post-operative outcomes due to the ability to obtain a relatively large sample size more quickly. Tracheostomy (13 per cent), cochlear implantation (11 per cent) and sleep-disordered breathing (9 per cent) also featured prominently. These conditions are associated with substantial lifestyle adjustments, complex care needs and critical parental decision-making, which align well with qualitative methodologies designed to capture lived experiences.

It is notable that no other single pathology featured in more than 6 per cent of studies. This concentration of qualitative research within a relatively small number of conditions suggests that while some high-impact areas are well represented, others remain comparatively underexplored. Though this does enable studies to build upon earlier research and facilitate comparison across different countries and environments, future research may benefit from seeking to redress the imbalance and ensure that the full breadth of paediatric otolaryngology conditions is explored through qualitative approaches.

### Methods of included studies

This review demonstrates clear patterns in the methodological and participant choices of qualitative research in paediatric otolaryngology. A substantial proportion of studies (13 per cent) employed more than one methodological approach, while over a third (34 per cent) drew data from multiple categories of participants. This overlap suggests that researchers often seek triangulation to enhance the validity of their findings, recognising that the experiences of different stakeholders and the strengths of varied qualitative methods can provide complementary perspectives.^17^

Interviews overall were by far the most common method (73 per cent), reflecting their well-established utility in healthcare research. The heavy skew towards semi-structured interviews (65 per cent) is striking, with 89 per cent of interview-based studies adopting this format. Semi-structured interviews offer a balance between consistency and flexibility, providing enough structure to ensure comparability across participants, while allowing scope to pursue unexpected, but relevant themes.^18^ This makes them especially valuable in paediatric ENT, where families and clinicians may introduce nuanced considerations beyond predefined researcher expectations. Numerous reviewed papers also used structured interviews as a modality for delivering survey-style questions. However, in some cases, it may be beneficial to consider utilising other qualitative interview methodologies that are currently underused. For instance, narrative interviewing can be considered to enable participants to produce richer, more detailed stories in their own words, minimising interviewer influence compared with semi-structured interviews.^19^

The overall reliance on interviews risks narrowing the methodological landscape. Questionnaires/surveys (22 per cent), social media analyses (7 per cent) and focus groups (6 per cent) were used much less frequently, despite each offering distinct advantages. Surveys can capture perspectives across larger samples at lower cost, facilitating breadth which in-depth interviews may sacrifice for depth. Focus groups, though logistically more demanding, encourage interaction between multiple participants, which generates insights into collective norms, shared experiences and group dynamics that may be obscured in individual interviews.^20^ Social media analysis, though still relatively novel in paediatric ENT, offers access to discourse which is less filtered and naturally occurring. This can be useful for capturing patient and caregiver voices outside clinical contexts.^21^ Broader adoption of these methods could therefore enrich the literature landscape and mitigate potential biases inherent in an interview-dominated evidence base.

The profile of research participants further reflects the field’s priorities. Families and primary caregivers were most frequently studied (62 per cent), which is unsurprising given their central role in decision-making, daily management of chronic conditions and interpretation of children’s needs. Particularly when considering the ethical and methodological challenges of direct paediatric participation, patients’ families often become an invaluable proxy through which patients’ perspectives can be better understood.^22^ Non-otolaryngologist healthcare professionals were also relatively well represented (27 per cent), perhaps due to the importance of multi-disciplinary input in paediatric otolaryngology (e.g. audiologists, speech therapists, specialist nurses, anaesthesiologists). Patients themselves were only explored as research subjects in 24 per cent of studies and otolaryngologists in 13 per cent. Though the relative under-representation of the former is easily explainable given the aforementioned challenges, this is not the case for the latter. Given their unique insights into clinical decision-making and system-level challenges, greater inclusion of surgeon perspectives could complement family and patient narratives.

### Purpose of research

The primary aims of the included studies were heterogeneous but coherent with the strengths of qualitative enquiry. The distributions mirror what we would expect, given the strengths of qualitative methods: generating rich, contextualised accounts of experience and meaning, exploring processes and relationships, and producing the content validity and user-centred insight needed to design interventions, measures and services.^29^^,^^30^

**Table 1.** Examples of papers coded by research purpose

Exploring barriers to access	*Barriers to timely diagnosis and treatment for children with hearing impairment in a southern Indian city: a qualitative study of parents and clinic staff.*^23^
Impact on families & carers	*Impact of COVID−19 on carers of children with tracheostomies.*^24^
Evaluating novel but existing tools & techniques	*Perceptions of otolaryngologists on single-entry models for managing wait times in community-based health care in Ontario: a qualitative study.*^25^
Developing novel tools & techniques	*The development of an interventional package on ‘receptive vocabulary’ for cochlear implanted children.*^26^
Examining stakeholder decision-making	*‘My Plate is Full’: reasons for declining a genetic evaluation of hearing loss.*^27^
Evaluating services	*A case study of enhanced clinical care enabled by Aboriginal health research: the Hearing, EAr health and Language Services (HEALS) project.*^28^

The most prevalent aim of included studies, exploring barriers to access (15 per cent), is a paradigmatic qualitative question. Identifying obstacles to care – whether practical (transport, cost), structural (referral pathways, appointment availability), cultural or informational – requires attention to how people experience the health system over time and in context. Qualitative methods are well-suited to capturing these nuanced contextual and social factors, which are often invisible in quantitative datasets.^31^

A substantial proportion of studies focused on the impact of illness and treatment on families and carers (12 per cent), reflecting the family-centred nature of paediatric ENT. Chronic conditions (hearing loss, recurrent tonsillitis, etc.) impose not only direct morbidity on the child, but also secondary burdens on parents, including disrupted sleep, workplace absenteeism and psychological stress. These factors, in turn, influence the ability of families to provide care for the affected child.

The prominence of studies aimed at evaluating new but existing tools and techniques (12 per cent) can be associated with that of developing outcome measures and other novel tools (10 per cent). These highlight another practical role for qualitative research: generating and validating content for patient-reported outcomes, decision aids and other instruments. US regulatory and methodological guidance explicitly recommends qualitative work (e.g. concept elicitation, cognitive interviewing) to establish content validity and ensure that items reflect patient priorities and language.^32^

Another prominent group of objectives was examining stakeholder decision-making (11 per cent), concerns (7 per cent) and expectations (3 per cent). These reflect the highly preference-sensitive nature of paediatric otolaryngology care. Parents’ anxieties about topics such as anaesthesia, complications of surgery and long-term outcomes often heavily influence the decisions that they make regarding care. Exploring these topics further highlights how families make decisions in ways beyond a numerical cost-benefit analysis and evaluate success differently from objective biomedical outcomes.

A smaller but important sub-set of studies focused on evaluating existing services (9 per cent), examining how current models of paediatric otolaryngology care perform in meeting patient and family needs. These evaluations often explored perceptions of care quality, continuity and accessibility, as well as how effectively services deliver intended outcomes. Through in-depth accounts from users and providers, qualitative methods illuminate why certain service models succeed or fail in practice, offering insight that can guide refinement, scaling or replacement of existing systems.

### Limitations

This review has several limitations. First, restriction to English-language publications may have excluded a substantial body of relevant research and introduced linguistic or cultural bias.^9^ Second, coding of study aims and methods required interpretative judgement; although an iterative process was adopted to minimise subjectivity, such decisions cannot be entirely free from researcher influence. Third, this was a descriptive mapping analysis rather than a full systematic synthesis. As such, findings identify patterns and gaps but do not provide deeper thematic analysis or critical appraisal of study quality.

## Conclusions

Qualitative research in paediatric otolaryngology has grown rapidly in recent years, dominated by work from the USA, Canada and the UK. The field has focused heavily on otology and laryngology, particularly on conditions such as hearing loss and tonsillectomy, which lend themselves to rich exploration of patient and family experiences. Semi-structured interviews with caregivers remain the dominant methodology and participant group, reflecting feasibility and appropriateness but limiting methodological diversity. Study aims are concentrated around access to care, family impact and decision-making, with relatively fewer efforts to guide research agendas or evaluate services. Future research should diversify methodological approaches, include under-represented stakeholders (including patients and surgeons) and expand to less-studied pathologies to build a more balanced and multidimensional understanding of paediatric otolaryngology care.

## Supporting information

10.1017/S0022215125104015.sm001 Mallis et al. supplementary material Mallis et al. supplementary material

## Supplementary material

The supplementary material for this article can be found at Lawson https://doi.org/10.1017/S0022215125104015.

## CRediT author statement

**Adam Mallis:** Conceptualisation, formal analysis, investigation, methodology, validation, visualisation, writing - original draft, writing - review and editing. **Angus Lawson:** Investigation, validation, writing - review and editing. **Jason Powell:** Conceptualisation, supervision, writing - review and editing.

## Appendix 1

1. Baqays A, Rashid M, Johannsen W, Seikaly H, El-Hakim H. What are parents’ perceptions related to barriers in diagnosing swallowing dysfunction in children? a grounded theory approach. BMJ Open 2021;11:e041591
2. Hall A, Pryce H, Bruce IA, Callery P, Lakhanpaul M, Schilder AGM. A mixed-methods study of the management of hearing loss associated with otitis media with effusion in children with Down syndrome. Clin Otolaryngol 2019;44:32–38
3. Heyward A, Hagerty K, Lichten L, Howell J, Tey CS, Dedhia K et al. The qualitative experiences of otolaryngologists with genetic services in pediatric hearing loss evaluation. J Community Genet 2023;14:377–385
4. Nilsen AH, Helvik A-S, Thorstensen WM, Austad B. “It is difficult for us to assess the severity!” a qualitative analysis of parents’ expectations to postoperative care after ventilation tube surgery. Int J Pediatr Otorhinolaryngol 2024;181:111941
5. Follent AM, Rumbach AF, Ward EC, Marshall J, Dodrill P, Lewindon P. Dysphagia and feeding difficulties post-pediatric ingestion injury: perspectives of the primary caregiver. Int J Pediatr Otorhinolaryngol 2017;103:20–28
6. Links AR, Tunkel DE, Boss EF. Stakeholder-engaged measure development for pediatric obstructive sleep-disordered breathing: the obstructive sleep-disordered breathing and adenotonsillectomy knowledge scale for parents. JAMA Otolaryngol Head Neck Surg 2017;143:46–54
7. Links AR, Callon W, Wasserman C, Beach MC, Ryan MA, Leu GR et al. Treatment recommendations to parents during pediatric tonsillectomy consultations: a mixed methods analysis of surgeon language. Patient Educ Couns 2021;104:1371–1379
8. Gorodzinsky AY, Hong P, Chorney JM. Parental knowledge in pediatric otolaryngology surgical consultations: a qualitative content analysis. Int J Pediatr Otorhinolaryngol 2015;79:1135–1139
9. Austad B, Nilsen AH, Thorstensen WM, Helvik A-S. Postoperative care for children after ventilation tube surgery: a qualitative study of parents’ experiences over time in Norway. Am J Otolaryngol 2024;45:104457
10. Ward B, Bavier R, Warren C, Yan J, Paskhover B. Qualitative evaluation of paediatric surgical otolaryngology content on YouTube. J Laryngol Otol 2020;1–3
11. McNeely BD, Fitzpatrick N, Leitmeyer K, Pauwels J, Chadha NK. Surgeon perspectives of three-dimensional endoscopy in paediatric otolaryngology: a qualitative study. Clin Otolaryngol 2023;48:920–924
12. Chitguppi C, Brar T. Do otolaryngology patients show gender preference when choosing a surgeon? a quantitative and qualitative analysis. Int Arch Otorhinolaryngol 2018;22:404–407
13. Haighton C, Watson RM, Wilson JA, Powell S. Caregiver acceptability of a UK trial for paediatric sleep disordered breathing: a qualitative interview study. Clin Otolaryngol 2024;49:254–257
14. Haighton C, Watson RM, Wilson JA, Powell S. Perspectives on paediatric sleep-disordered breathing in the UK: a qualitative study. J Laryngol Otol 2022;136:520–526
15. Mahomva C, Harris S, Seebran N, Mudge B, Catlin B, Davies L. Improving access to school based education for South African children in rural areas who have a tracheostomy: a case series and recommendations. Int J Pediatr Otorhinolaryngol 2017;92:186–192
16. Young C, Gunasekera H, Kong K, Purcell A, Muthayya S, Vincent F et al. A case study of enhanced clinical care enabled by Aboriginal health research: the Hearing, EAr health and Language Services (HEALS) project. Aust N Z J Public Health 2016;40:523–528
17. Long CG, Smith DH. Parental pressure for tonsillectomy: attitudes and knowledge of parents accompanying their children to an ear, nose and throat clinic. Psychol Med 1985;15:689–693
18. Howe CJ, Lewis B, Edmondson S. Barriers and facilitators to implementing health literacy practices in a pediatric ENT clinic: a mixed-methods study. J Nurs Care Qual 2024;39:106–113
19. Massey CJ, Asokan A, Tietbohl C, Morris M, Ramakrishnan VR. Otolaryngologist perceptions of AI-based sinus CT interpretation. Am J Otolaryngol 2023;44:103932
20. Heward E, Lunn J, Birkenshaw-Dempsey J, Molloy J, Isba R, Ashcroft DM et al. Exploring the burden of paediatric acute otitis media with discharge in the UK: a qualitative study. BMJ Paediatr Open 2024;8:e003012
21. Maguire E, Hong P, Ritchie K, Meier J, Archibald K, Chorney J. Decision aid prototype development for parents considering adenotonsillectomy for their children with sleep disordered breathing. J Otolaryngol Head Neck Surg 2016;45:57
22. Maki-Torkko E, Sorri M, Jarvelin MR. More education in paediatric audiology needed for child welfare clinic nurses and doctors. Public Health 1997;111:93–96
23. Moradi-Joo E, Barouni M, Vali L, Mahmoudian S. Qualitative analysis of newborn hearing screening program in Iran. Med J Islam Repub Iran 2024;38:23
24. Na E, Toupin-April K, Olds J, Noll D, Fitzpatrick EM. Cochlear implant decision making for children with residual hearing: perspectives of practitioners. Am J Audiol 2023;32:334–346
25. Na E, Toupin-April K, Olds J, Noll D, Fitzpatrick EM. Cochlear implant decision-making for children with residual hearing: perspectives of parents. Cochlear Implants Int 2023;24:301–310
26. Boss EF, Links AR, Saxton R, Cheng TL, Beach MC. Parent experience of care and decision making for children who snore. JAMA Otolaryngol Head Neck Surg 2017;143:218–225
27. Boss EF, Links AR, Saxton R, Cheng TL, Beach MC. Physician perspectives on decision making for treatment of pediatric sleep-disordered breathing. Clin Pediatr (Phila) 2017;56:993–1000
28. Huang EY, Hairston TK, Walsh J, Ballard ME, Boss EF, Jenks CM. Evaluation of parental perspectives and concerns about pediatric cochlear implantation: a social media analysis. Otol Neurotol 2023;44:e715–e721
29. Lin FR, Ceh K, Bervinchak D, Riley A, Miech R, Niparko JK. Development of a communicative performance scale for pediatric cochlear implantation. Ear Hear 2007;28:703–712
30. Brown G, Warrington N, Ulph F, Booth N, Harvey K, James R et al. Exploring NICU nurses’ views of a novel genetic point-of-care test identifying neonates at risk of antibiotic-induced ototoxicity: a qualitative study. J Adv Nurs 2024;80:3359–3370
31. Cejas I, Coto J, Sarangoulis C, Hoffman MF, Quittner AL. Development and validation of a parenting stress module for parents of children using cochlear implants. J Pediatr Psychol 2022;47:785–794
32. Sambah I, Zhao F, El-Lishani R. The professional’s experience with causes of delay in the diagnosis and management of children with a congenital hearing loss in Libya. Int J Pediatr Otorhinolaryngol 2020;128:109687
33. Vlastos IM, Hajiioannou J, Houlakis M. Otitis media with effusion: what parents want to know. J Laryngol Otol 2008;122:21–24
34. Elpers J, Lester C, Shinn JB, Bush ML. Rural family perspectives and experiences with early infant hearing detection and intervention: a qualitative study. J Community Health 2016;41:226–233
35. Longard J, Twycross A, Williams AM, Hong P, Chorney J. Parents’ experiences of managing their child’s postoperative pain at home: an exploratory qualitative study. J Clin Nurs 2016;25:2619–2628
36. Shapiro J, Perlmutter J, Axelrod C, Bandargal S, Pundaky G, Levy BB et al. Perceptions of otolaryngologists on single-entry models for managing wait times in community-based health care in Ontario: a qualitative study. J Otolaryngol Head Neck Surg 2025;54:19160216251336682
37. Sherman J, Zalzal H, Bower K. Equitable care for children with a tracheostomy: addressing challenges and seeking systemic solutions. Health Expect 2024;27:e14158
38. Skirko JR, Pollard SH, Slager S, Hung M, Weir C. Family experience with Pierre Robin sequence: a qualitative study. Cleft Palate Craniofac J 2020;57:736–745
39. Balakrishnan K, Edwards TC, Perkins JA. Functional and symptom impacts of pediatric head and neck lymphatic malformations: developing a patient-derived instrument. Otolaryngol Head Neck Surg 2012;147:925–931
40. Gfeller K, Mallalieu R. Psychosocial and auditory factors that influence successful music-based auditory training in pediatric cochlear implant recipients. Front Hum Neurosci 2023;17:1308712
41. Jaradeh K, Liao EN, Chehab LZ, Tebb KP, Florentine MM, Bellfort-Salinas S et al. Understanding barriers to timely diagnosis and intervention among immigrant children with hearing loss. Otolaryngol Head Neck Surg 2023;169:710–718
42. Chua K-P, Thorne MC, Brummett CM, DeJonckheere M. Surgeons’ perspectives on changing the default number of doses for opioid prescriptions in electronic health record systems. JAMA Netw Open 2023;6:e2315633
43. Barr L, Thibeault SL, Muntz H, de Serres L. Quality of life in children with velopharyngeal insufficiency. Arch Otolaryngol Head Neck Surg 2007;133:224–229
44. Booth L, Pauwels J, Chadha NK, Felton M. “A lonely time for deaf and hard of hearing kids”: a qualitative study of the impact of pandemic precautions on classroom communication for adolescents with hearing loss. Int J Pediatr Otorhinolaryngol 2024;181:111989
45. DiNoto L, Frankel A, Wheaton T, Smith D, Buholtz K, Dadiz R et al. Navigating a new normal: a mixed-methods study of the pediatric tracheostomy parent-caregiver experience. Children (Basel) 2025;12:956
46. Fraser L, Montgomery J, James H, Wynne DM, MacGregor FB, Clement WA et al. Validation of a family-centred outcome questionnaire for pinnaplasty: a cross-sectional pilot study. Clin Otolaryngol 2016;41:472–480
47. Monshizadeh L, Vameghi R, Sajedi F, Yadegari F, Rahimi M, Hashemi SB. The development of an interventional package on “receptive vocabulary” for cochlear implanted children. Iran J Child Neurol 2019;13:113–123
48. Claus LE, Amos JM, Links AR, Beach MC, Boss EF. Surgeon information-sharing, parent verbal engagement, and parent knowledge of pediatric adenotonsillectomy. Otolaryngol Head Neck Surg 2024;170:552–559
49. Fisher LM, Martinez AS, Richmond FJ, Krieger MD, Wilkinson E, Eisenberg LS. Assessing the benefit-risk profile for pediatric implantable auditory prostheses. Ther Innov Regul Sci 2018;52:669–679
50. Lindburg M, Ead B, Jeffe DB, Lieu JEC. Hearing loss-related issues affecting quality of life in preschool children. Otolaryngol Head Neck Surg 2021;164:1322–1329
51. Moradi M, Fallahi-Khoshknab M, Dalvandi A, Farhadi M, Maddah SSB, Mohammadi E. Family and rehabilitation of children with cochlear implant: a qualitative study. Florence Nightingale J Nurs 2022;30:18–24
52. Purcell M, Longard J, Chorney J, Hong P. Parents’ experiences managing their child’s complicated postoperative recovery. Int J Pediatr Otorhinolaryngol 2018;106:50–54
53. Koenigs MB, Behzadpour HK, Harrington CB, Prado L, Gorelik D, Woolman K et al. Barriers to pediatric osseointegrated bone-conduction hearing devices. Otol Neurotol 2022;43:e590–e596
54. McCormick ME, Ward E, Roberson DW, Shah RK, Stachler RJ, Brenner MJ. Life after tracheostomy: patient and family perspectives on teaching, transitions, and multidisciplinary teams. Otolaryngol Head Neck Surg 2015;153:914–920
55. Lesperance MM, Winkler E, Melendez TL, Yashar BM. “My plate is full”: reasons for declining a genetic evaluation of hearing loss. J Genet Couns 2018;27:597–607
56. Alam MN, Munjal S, Sharma A, Panda N, Banumathy N. Parental expectation and perception of CI benefits in their implanted wards. Indian J Otolaryngol Head Neck Surg 2019;71:1153–1156
57. Braden MN, van Leer E. Effect of MP4 therapy videos on adherence to voice therapy home practice in children with dysphonia. J Voice 2017;31:114.e17–114.e23
58. Braden MN, van Leer E, McConville K, Blakeslee SDM. Patient, parent, and speech-language pathologists’ perceptions of pediatric voice therapy through interviews. Am J Speech Lang Pathol 2018;27:1385–1404
59. Xanthopoulos MS, Nelson MN, Eriksen W, Barg FK, Byars KC, Ishman SL et al. Caregiver experiences helping children with Down syndrome use positive airway pressure to treat obstructive sleep apnea. Sleep Med 2023;107:179–186
60. Farias N, Rose-Davis B, Hong P, Wozney L. An automated text messaging system (Tonsil-Text-To-Me) to improve tonsillectomy perioperative experience: exploratory qualitative usability and feasibility study. JMIR Perioper Med 2020;3:e14601
61. Hall N, Rousseau N, Hamilton DW, Simpson AJ, Powell S, Brodlie M et al. Providing care for children with tracheostomies: a qualitative interview study with parents and health professionals. BMJ Open 2023;13:e065698
62. Hall N, Rousseau N, Hamilton DW, Simpson AJ, Powell S, Brodlie M et al. Impact of COVID-19 on carers of children with tracheostomies. Arch Dis Child 2022;107:e23
63. Prabhu N, MacNevin W, Wheelock M, Hong P, Bezuhly M. Understanding child anxiety before otoplasty: a qualitative study. Int J Pediatr Otorhinolaryngol 2020;139:110489
64. Rashidi N, Lindeborg MM, Stephans J, Bellfort-Salinas S, Naugle K, Wong MA et al. Understanding and improving pediatric hearing care navigation: a human-centered design approach. Otolaryngol Head Neck Surg 2025;172:1418–1426
65. Vukkadala N, Giridhar SBP, Okumura MJ, Chan DK. Seeking equilibrium: the experiences of parents of infants and toddlers who are deaf/hard-of-hearing. J Pediatr Rehabil Med 2019;12:11–20
66. Rossi NA, French KR, Evans CL, Ohlstein JF, Neve LD, Daram S et al. Trending tubes: a social media analysis of tympanostomy tubes in children. OTO Open 2022;6:2473974X221086964
67. Chadha NK, Allegro J, Barton M, Hawkes M, Harlock H, Campisi P. The quality of life and health utility burden of recurrent respiratory papillomatosis in children. Otolaryngol Head Neck Surg 2010;143:685–690
68. Chadha NK, Gordon KA, James AL, Papsin BC. Tinnitus is prevalent in children with cochlear implants. Int J Pediatr Otorhinolaryngol 2009;73:671–675
69. Gilbey P. Qualitative analysis of parents’ experience with receiving the news of the detection of their child’s hearing loss. Int J Pediatr Otorhinolaryngol 2010;74:265–270
70. Purcell PL, Jones-Goodrich R, Wisneski M, Edwards TC, Sie KCY. Hearing devices for children with unilateral hearing loss: patient- and parent-reported perspectives. Int J Pediatr Otorhinolaryngol 2016;90:43–48
71. Purcell PL, Edwards TC, Wisneski M, Chan DK, Ou H, Horn DL et al. Unilateral hearing loss in youth: development of candidate items for a condition-specific validated instrument. Otolaryngol Head Neck Surg 2018;159:1043–1050
72. Pecha PP, Jungbauer WN, Ruggiero KJ, Nietert P, Melvin CL, Ford ME. Parental experiences with access to care for obstructive sleep-disordered breathing: a qualitative study. Otolaryngol Head Neck Surg 2023;169:1319–1328
73. Vo QT, Pham D, Choi KJ, Nguyen UTT, Le L, Shanewise T et al. Solar-powered hearing aids for children with impaired hearing in Vietnam: a pilot study. Paediatr Int Child Health 2018;38:40–45
74. Begley R, Kanagasingam Y, Chan C, Perera C, Vandeleur M, Paddle P. Demonstration of accuracy and feasibility of remotely delivered oximetry: a blinded, controlled, real-world study of regional/rural children with obstructive sleep apnoea. Healthcare (Basel) 2023;11:278
75. Fahy R, Corbett M, Keogh I. Improving peri-operative psychosocial interventions for children with autism spectrum disorder undergoing ENT procedures. J Laryngol Otol 2020;1–7
76. Stewart R, Gallagher D, Leyden P. Diagnosis and management of conductive hearing loss in children with trisomy 21. J Paediatr Child Health 2018;54:1242–1245
77. Asthana S, Hassan MT, Hassan O, Alkhalili S, Keshwani A, Gonzalez F et al. Social determinants of health and effectiveness of social work support in a pediatric aerodigestive program. Int J Pediatr Otorhinolaryngol 2025;195:112459
78. DeForte S, Sezgin E, Huefner J, Lucius S, Luna J, Satyapriya AA et al. Usability of a mobile app for improving literacy in children with hearing impairment: focus group study. JMIR Hum Factors 2020;7:e16310
79. Gong S, Wang X, Wang Y, Qu Y, Tang C, Yu Q et al. A descriptive qualitative study of home care experiences in parents of children with tracheostomies. J Pediatr Nurs 2019;45:7–12
80. Polubothu S, Blackmore KJ, Kubba H. Outcomes of surgery for laryngotracheal stenosis—the parents perspective. Int J Pediatr Otorhinolaryngol 2011;75:425–429
81. Grond SE, Kallies G, McCormick ME. Parental and provider perspectives on social media about ankyloglossia. Int J Pediatr Otorhinolaryngol 2021;146:110741
82. Olsson SE, Schmitz JF, Huang AE, Murray AD. A descriptive analysis of otolaryngology presence on the social media platform TikTok. Laryngoscope Investig Otolaryngol 2023;8:1516–1521
83. Merugumala SV, Pothula V, Cooper M. Barriers to timely diagnosis and treatment for children with hearing impairment in a southern Indian city: a qualitative study of parents and clinic staff. Int J Audiol 2017;56:733–739
84. Hairston TK, Links AR, Harris V, Tunkel DE, Walsh J, Beach MC et al. Evaluation of parental perspectives and concerns about pediatric tonsillectomy in social media. JAMA Otolaryngol Head Neck Surg 2019;145:45–52
85. Khan U, Luther E, Cassidy CE, Boss E, Meister KD, Bohm M et al. The barriers and facilitators of shared decision making in pediatric otolaryngology: a qualitative study. Otolaryngol Head Neck Surg 2025;172:273–282
86. Findlen UM, Malhotra PS, Adunka OF. Parent perspectives on multidisciplinary pediatric hearing healthcare. Int J Pediatr Otorhinolaryngol 2019;116:141–146
87. Gkiousias V, Butler CC, Shepherd V, Kilgour JM, Waldron C-A, Thomas-Jones E et al. Parental perceptions and understanding of information provision, management options and factors influencing the decision-making process in the treatment of children with glue ear. Int J Pediatr Otorhinolaryngol 2016;89:6–12
88. Sung V, Ching TYC, Smith L, Marnane V, Saetre-Turner M, King A et al. Mild matters: trial learnings and importance of community engagement in research for early identified bilateral mild hearing loss. Front Pediatr 2023;11:1197739
89. Leeper WR, Haut ER, Pandian V, Nakka S, Dodd-O J, Bhatti N et al. Multidisciplinary difficult airway course: an essential educational component of a hospital-wide difficult airway response program. J Surg Educ 2018;75:1264–1275
