# Supplementary material for: A systematic mapping review of qualitative research in paediatric otolaryngology
Source: J Laryngol Otol. 2026 Mar;140(3):286–97. doi: 10.1017/S0022215125104015 (PMC13058413; doi:10.1017/S0022215125104015)
Supplement: Mallis et al. supplementary material [file S0022215125104015sup001.docx]

1. Baqays A, Rashid M, Johannsen W, Seikaly H, El-Hakim H. What are parents’ perceptions related to barriers in diagnosing swallowing dysfunction in children? a grounded theory approach. BMJ Open 2021;11:e041591
2. Hall A, Pryce H, Bruce IA, Callery P, Lakhanpaul M, Schilder AGM. A mixed-methods study of the management of hearing loss associated with otitis media with effusion in children with Down syndrome. Clin Otolaryngol 2019;44:32–38
3. Heyward A, Hagerty K, Lichten L, Howell J, Tey CS, Dedhia K et al. The qualitative experiences of otolaryngologists with genetic services in pediatric hearing loss evaluation. J Community Genet 2023;14:377–385
4. Nilsen AH, Helvik A-S, Thorstensen WM, Austad B. “It is difficult for us to assess the severity!” a qualitative analysis of parents’ expectations to postoperative care after ventilation tube surgery. Int J Pediatr Otorhinolaryngol 2024;181:111941
5. Follent AM, Rumbach AF, Ward EC, Marshall J, Dodrill P, Lewindon P. Dysphagia and feeding difficulties post-pediatric ingestion injury: perspectives of the primary caregiver. Int J Pediatr Otorhinolaryngol 2017;103:20–28
6. Links AR, Tunkel DE, Boss EF. Stakeholder-engaged measure development for pediatric obstructive sleep-disordered breathing: the obstructive sleep-disordered breathing and adenotonsillectomy knowledge scale for parents. JAMA Otolaryngol Head Neck Surg 2017;143:46–54
7. Links AR, Callon W, Wasserman C, Beach MC, Ryan MA, Leu GR et al. Treatment recommendations to parents during pediatric tonsillectomy consultations: a mixed methods analysis of surgeon language. Patient Educ Couns 2021;104:1371–1379
8. Gorodzinsky AY, Hong P, Chorney JM. Parental knowledge in pediatric otolaryngology surgical consultations: a qualitative content analysis. Int J Pediatr Otorhinolaryngol 2015;79:1135–1139
9. Austad B, Nilsen AH, Thorstensen WM, Helvik A-S. Postoperative care for children after ventilation tube surgery: a qualitative study of parents’ experiences over time in Norway. Am J Otolaryngol 2024;45:104457
10. Ward B, Bavier R, Warren C, Yan J, Paskhover B. Qualitative evaluation of paediatric surgical otolaryngology content on YouTube. J Laryngol Otol 2020;1–3
11. McNeely BD, Fitzpatrick N, Leitmeyer K, Pauwels J, Chadha NK. Surgeon perspectives of three-dimensional endoscopy in paediatric otolaryngology: a qualitative study. Clin Otolaryngol 2023;48:920–924
12. Chitguppi C, Brar T. Do otolaryngology patients show gender preference when choosing a surgeon? a quantitative and qualitative analysis. Int Arch Otorhinolaryngol 2018;22:404–407
13. Haighton C, Watson RM, Wilson JA, Powell S. Caregiver acceptability of a UK trial for paediatric sleep disordered breathing: a qualitative interview study. Clin Otolaryngol 2024;49:254–257
14. Haighton C, Watson RM, Wilson JA, Powell S. Perspectives on paediatric sleep-disordered breathing in the UK: a qualitative study. J Laryngol Otol 2022;136:520–526
15. Mahomva C, Harris S, Seebran N, Mudge B, Catlin B, Davies L. Improving access to school based education for South African children in rural areas who have a tracheostomy: a case series and recommendations. Int J Pediatr Otorhinolaryngol 2017;92:186–192
16. Young C, Gunasekera H, Kong K, Purcell A, Muthayya S, Vincent F et al. A case study of enhanced clinical care enabled by Aboriginal health research: the Hearing, EAr health and Language Services (HEALS) project. Aust N Z J Public Health 2016;40:523–528
17. Long CG, Smith DH. Parental pressure for tonsillectomy: attitudes and knowledge of parents accompanying their children to an ear, nose and throat clinic. Psychol Med 1985;15:689–693
18. Howe CJ, Lewis B, Edmondson S. Barriers and facilitators to implementing health literacy practices in a pediatric ENT clinic: a mixed-methods study. J Nurs Care Qual 2024;39:106–113
19. Massey CJ, Asokan A, Tietbohl C, Morris M, Ramakrishnan VR. Otolaryngologist perceptions of AI-based sinus CT interpretation. Am J Otolaryngol 2023;44:103932
20. Heward E, Lunn J, Birkenshaw-Dempsey J, Molloy J, Isba R, Ashcroft DM et al. Exploring the burden of paediatric acute otitis media with discharge in the UK: a qualitative study. BMJ Paediatr Open 2024;8:e003012
21. Maguire E, Hong P, Ritchie K, Meier J, Archibald K, Chorney J. Decision aid prototype development for parents considering adenotonsillectomy for their children with sleep disordered breathing. J Otolaryngol Head Neck Surg 2016;45:57
22. Maki-Torkko E, Sorri M, Jarvelin MR. More education in paediatric audiology needed for child welfare clinic nurses and doctors. Public Health 1997;111:93–96
23. Moradi-Joo E, Barouni M, Vali L, Mahmoudian S. Qualitative analysis of newborn hearing screening program in Iran. Med J Islam Repub Iran 2024;38:23
24. Na E, Toupin-April K, Olds J, Noll D, Fitzpatrick EM. Cochlear implant decision making for children with residual hearing: perspectives of practitioners. Am J Audiol 2023;32:334–346
25. Na E, Toupin-April K, Olds J, Noll D, Fitzpatrick EM. Cochlear implant decision-making for children with residual hearing: perspectives of parents. Cochlear Implants Int 2023;24:301–310
26. Boss EF, Links AR, Saxton R, Cheng TL, Beach MC. Parent experience of care and decision making for children who snore. JAMA Otolaryngol Head Neck Surg 2017;143:218–225
27. Boss EF, Links AR, Saxton R, Cheng TL, Beach MC. Physician perspectives on decision making for treatment of pediatric sleep-disordered breathing. Clin Pediatr (Phila) 2017;56:993–1000
28. Huang EY, Hairston TK, Walsh J, Ballard ME, Boss EF, Jenks CM. Evaluation of parental perspectives and concerns about pediatric cochlear implantation: a social media analysis. Otol Neurotol 2023;44:e715–e721
29. Lin FR, Ceh K, Bervinchak D, Riley A, Miech R, Niparko JK. Development of a communicative performance scale for pediatric cochlear implantation. Ear Hear 2007;28:703–712
30. Brown G, Warrington N, Ulph F, Booth N, Harvey K, James R et al. Exploring NICU nurses’ views of a novel genetic point-of-care test identifying neonates at risk of antibiotic-induced ototoxicity: a qualitative study. J Adv Nurs 2024;80:3359–3370
31. Cejas I, Coto J, Sarangoulis C, Hoffman MF, Quittner AL. Development and validation of a parenting stress module for parents of children using cochlear implants. J Pediatr Psychol 2022;47:785–794
32. Sambah I, Zhao F, El-Lishani R. The professional’s experience with causes of delay in the diagnosis and management of children with a congenital hearing loss in Libya. Int J Pediatr Otorhinolaryngol 2020;128:109687
33. Vlastos IM, Hajiioannou J, Houlakis M. Otitis media with effusion: what parents want to know. J Laryngol Otol 2008;122:21–24
34. Elpers J, Lester C, Shinn JB, Bush ML. Rural family perspectives and experiences with early infant hearing detection and intervention: a qualitative study. J Community Health 2016;41:226–233
35. Longard J, Twycross A, Williams AM, Hong P, Chorney J. Parents’ experiences of managing their child’s postoperative pain at home: an exploratory qualitative study. J Clin Nurs 2016;25:2619–2628
36. Shapiro J, Perlmutter J, Axelrod C, Bandargal S, Pundaky G, Levy BB et al. Perceptions of otolaryngologists on single-entry models for managing wait times in community-based health care in Ontario: a qualitative study. J Otolaryngol Head Neck Surg 2025;54:19160216251336682
37. Sherman J, Zalzal H, Bower K. Equitable care for children with a tracheostomy: addressing challenges and seeking systemic solutions. Health Expect 2024;27:e14158
38. Skirko JR, Pollard SH, Slager S, Hung M, Weir C. Family experience with Pierre Robin sequence: a qualitative study. Cleft Palate Craniofac J 2020;57:736–745
39. Balakrishnan K, Edwards TC, Perkins JA. Functional and symptom impacts of pediatric head and neck lymphatic malformations: developing a patient-derived instrument. Otolaryngol Head Neck Surg 2012;147:925–931
40. Gfeller K, Mallalieu R. Psychosocial and auditory factors that influence successful music-based auditory training in pediatric cochlear implant recipients. Front Hum Neurosci 2023;17:1308712
41. Jaradeh K, Liao EN, Chehab LZ, Tebb KP, Florentine MM, Bellfort-Salinas S et al. Understanding barriers to timely diagnosis and intervention among immigrant children with hearing loss. Otolaryngol Head Neck Surg 2023;169:710–718
42. Chua K-P, Thorne MC, Brummett CM, DeJonckheere M. Surgeons’ perspectives on changing the default number of doses for opioid prescriptions in electronic health record systems. JAMA Netw Open 2023;6:e2315633
43. Barr L, Thibeault SL, Muntz H, de Serres L. Quality of life in children with velopharyngeal insufficiency. Arch Otolaryngol Head Neck Surg 2007;133:224–229
44. Booth L, Pauwels J, Chadha NK, Felton M. “A lonely time for deaf and hard of hearing kids”: a qualitative study of the impact of pandemic precautions on classroom communication for adolescents with hearing loss. Int J Pediatr Otorhinolaryngol 2024;181:111989
45. DiNoto L, Frankel A, Wheaton T, Smith D, Buholtz K, Dadiz R et al. Navigating a new normal: a mixed-methods study of the pediatric tracheostomy parent-caregiver experience. Children (Basel) 2025;12:956
46. Fraser L, Montgomery J, James H, Wynne DM, MacGregor FB, Clement WA et al. Validation of a family-centred outcome questionnaire for pinnaplasty: a cross-sectional pilot study. Clin Otolaryngol 2016;41:472–480
47. Monshizadeh L, Vameghi R, Sajedi F, Yadegari F, Rahimi M, Hashemi SB. The development of an interventional package on “receptive vocabulary” for cochlear implanted children. Iran J Child Neurol 2019;13:113–123
48. Claus LE, Amos JM, Links AR, Beach MC, Boss EF. Surgeon information-sharing, parent verbal engagement, and parent knowledge of pediatric adenotonsillectomy. Otolaryngol Head Neck Surg 2024;170:552–559
49. Fisher LM, Martinez AS, Richmond FJ, Krieger MD, Wilkinson E, Eisenberg LS. Assessing the benefit-risk profile for pediatric implantable auditory prostheses. Ther Innov Regul Sci 2018;52:669–679
50. Lindburg M, Ead B, Jeffe DB, Lieu JEC. Hearing loss-related issues affecting quality of life in preschool children. Otolaryngol Head Neck Surg 2021;164:1322–1329
51. Moradi M, Fallahi-Khoshknab M, Dalvandi A, Farhadi M, Maddah SSB, Mohammadi E. Family and rehabilitation of children with cochlear implant: a qualitative study. Florence Nightingale J Nurs 2022;30:18–24
52. Purcell M, Longard J, Chorney J, Hong P. Parents’ experiences managing their child’s complicated postoperative recovery. Int J Pediatr Otorhinolaryngol 2018;106:50–54
53. Koenigs MB, Behzadpour HK, Harrington CB, Prado L, Gorelik D, Woolman K et al. Barriers to pediatric osseointegrated bone-conduction hearing devices. Otol Neurotol 2022;43:e590–e596
54. McCormick ME, Ward E, Roberson DW, Shah RK, Stachler RJ, Brenner MJ. Life after tracheostomy: patient and family perspectives on teaching, transitions, and multidisciplinary teams. Otolaryngol Head Neck Surg 2015;153:914–920
55. Lesperance MM, Winkler E, Melendez TL, Yashar BM. “My plate is full”: reasons for declining a genetic evaluation of hearing loss. J Genet Couns 2018;27:597–607
56. Alam MN, Munjal S, Sharma A, Panda N, Banumathy N. Parental expectation and perception of CI benefits in their implanted wards. Indian J Otolaryngol Head Neck Surg 2019;71:1153–1156
57. Braden MN, van Leer E. Effect of MP4 therapy videos on adherence to voice therapy home practice in children with dysphonia. J Voice 2017;31:114.e17–114.e23
58. Braden MN, van Leer E, McConville K, Blakeslee SDM. Patient, parent, and speech-language pathologists’ perceptions of pediatric voice therapy through interviews. Am J Speech Lang Pathol 2018;27:1385–1404
59. Xanthopoulos MS, Nelson MN, Eriksen W, Barg FK, Byars KC, Ishman SL et al. Caregiver experiences helping children with Down syndrome use positive airway pressure to treat obstructive sleep apnea. Sleep Med 2023;107:179–186
60. Farias N, Rose-Davis B, Hong P, Wozney L. An automated text messaging system (Tonsil-Text-To-Me) to improve tonsillectomy perioperative experience: exploratory qualitative usability and feasibility study. JMIR Perioper Med 2020;3:e14601
61. Hall N, Rousseau N, Hamilton DW, Simpson AJ, Powell S, Brodlie M et al. Providing care for children with tracheostomies: a qualitative interview study with parents and health professionals. BMJ Open 2023;13:e065698
62. Hall N, Rousseau N, Hamilton DW, Simpson AJ, Powell S, Brodlie M et al. Impact of COVID-19 on carers of children with tracheostomies. Arch Dis Child 2022;107:e23
63. Prabhu N, MacNevin W, Wheelock M, Hong P, Bezuhly M. Understanding child anxiety before otoplasty: a qualitative study. Int J Pediatr Otorhinolaryngol 2020;139:110489
64. Rashidi N, Lindeborg MM, Stephans J, Bellfort-Salinas S, Naugle K, Wong MA et al. Understanding and improving pediatric hearing care navigation: a human-centered design approach. Otolaryngol Head Neck Surg 2025;172:1418–1426
65. Vukkadala N, Giridhar SBP, Okumura MJ, Chan DK. Seeking equilibrium: the experiences of parents of infants and toddlers who are deaf/hard-of-hearing. J Pediatr Rehabil Med 2019;12:11–20
66. Rossi NA, French KR, Evans CL, Ohlstein JF, Neve LD, Daram S et al. Trending tubes: a social media analysis of tympanostomy tubes in children. OTO Open 2022;6:2473974X221086964
67. Chadha NK, Allegro J, Barton M, Hawkes M, Harlock H, Campisi P. The quality of life and health utility burden of recurrent respiratory papillomatosis in children. Otolaryngol Head Neck Surg 2010;143:685–690
68. Chadha NK, Gordon KA, James AL, Papsin BC. Tinnitus is prevalent in children with cochlear implants. Int J Pediatr Otorhinolaryngol 2009;73:671–675
69. Gilbey P. Qualitative analysis of parents’ experience with receiving the news of the detection of their child’s hearing loss. Int J Pediatr Otorhinolaryngol 2010;74:265–270
70. Purcell PL, Jones-Goodrich R, Wisneski M, Edwards TC, Sie KCY. Hearing devices for children with unilateral hearing loss: patient- and parent-reported perspectives. Int J Pediatr Otorhinolaryngol 2016;90:43–48
71. Purcell PL, Edwards TC, Wisneski M, Chan DK, Ou H, Horn DL et al. Unilateral hearing loss in youth: development of candidate items for a condition-specific validated instrument. Otolaryngol Head Neck Surg 2018;159:1043–1050
72. Pecha PP, Jungbauer WN, Ruggiero KJ, Nietert P, Melvin CL, Ford ME. Parental experiences with access to care for obstructive sleep-disordered breathing: a qualitative study. Otolaryngol Head Neck Surg 2023;169:1319–1328
73. Vo QT, Pham D, Choi KJ, Nguyen UTT, Le L, Shanewise T et al. Solar-powered hearing aids for children with impaired hearing in Vietnam: a pilot study. Paediatr Int Child Health 2018;38:40–45
74. Begley R, Kanagasingam Y, Chan C, Perera C, Vandeleur M, Paddle P. Demonstration of accuracy and feasibility of remotely delivered oximetry: a blinded, controlled, real-world study of regional/rural children with obstructive sleep apnoea. Healthcare (Basel) 2023;11:278
75. Fahy R, Corbett M, Keogh I. Improving peri-operative psychosocial interventions for children with autism spectrum disorder undergoing ENT procedures. J Laryngol Otol 2020;1–7
76. Stewart R, Gallagher D, Leyden P. Diagnosis and management of conductive hearing loss in children with trisomy 21. J Paediatr Child Health 2018;54:1242–1245
77. Asthana S, Hassan MT, Hassan O, Alkhalili S, Keshwani A, Gonzalez F et al. Social determinants of health and effectiveness of social work support in a pediatric aerodigestive program. Int J Pediatr Otorhinolaryngol 2025;195:112459
78. DeForte S, Sezgin E, Huefner J, Lucius S, Luna J, Satyapriya AA et al. Usability of a mobile app for improving literacy in children with hearing impairment: focus group study. JMIR Hum Factors 2020;7:e16310
79. Gong S, Wang X, Wang Y, Qu Y, Tang C, Yu Q et al. A descriptive qualitative study of home care experiences in parents of children with tracheostomies. J Pediatr Nurs 2019;45:7–12
80. Polubothu S, Blackmore KJ, Kubba H. Outcomes of surgery for laryngotracheal stenosis—the parents perspective. Int J Pediatr Otorhinolaryngol 2011;75:425–429
81. Grond SE, Kallies G, McCormick ME. Parental and provider perspectives on social media about ankyloglossia. Int J Pediatr Otorhinolaryngol 2021;146:110741
82. Olsson SE, Schmitz JF, Huang AE, Murray AD. A descriptive analysis of otolaryngology presence on the social media platform TikTok. Laryngoscope Investig Otolaryngol 2023;8:1516–1521
83. Merugumala SV, Pothula V, Cooper M. Barriers to timely diagnosis and treatment for children with hearing impairment in a southern Indian city: a qualitative study of parents and clinic staff. Int J Audiol 2017;56:733–739
84. Hairston TK, Links AR, Harris V, Tunkel DE, Walsh J, Beach MC et al. Evaluation of parental perspectives and concerns about pediatric tonsillectomy in social media. JAMA Otolaryngol Head Neck Surg 2019;145:45–52
85. Khan U, Luther E, Cassidy CE, Boss E, Meister KD, Bohm M et al. The barriers and facilitators of shared decision making in pediatric otolaryngology: a qualitative study. Otolaryngol Head Neck Surg 2025;172:273–282
86. Findlen UM, Malhotra PS, Adunka OF. Parent perspectives on multidisciplinary pediatric hearing healthcare. Int J Pediatr Otorhinolaryngol 2019;116:141–146
87. Gkiousias V, Butler CC, Shepherd V, Kilgour JM, Waldron C-A, Thomas-Jones E et al. Parental perceptions and understanding of information provision, management options and factors influencing the decision-making process in the treatment of children with glue ear. Int J Pediatr Otorhinolaryngol 2016;89:6–12
88. Sung V, Ching TYC, Smith L, Marnane V, Saetre-Turner M, King A et al. Mild matters: trial learnings and importance of community engagement in research for early identified bilateral mild hearing loss. Front Pediatr 2023;11:1197739
89. Leeper WR, Haut ER, Pandian V, Nakka S, Dodd-O J, Bhatti N et al. Multidisciplinary difficult airway course: an essential educational component of a hospital-wide difficult airway response program. J Surg Educ 2018;75:1264–1275
